# An Ugly Buddha Fixed an Ugly Woman and Made Her a Beauty

**DOI:** 10.1055/a-2067-5563

**Published:** 2023-08-02

**Authors:** Kun Hwang

**Affiliations:** 1 Department of Plastic Surgery, Armed Forces Capital Hospital, Bundang-gu, Seongnam-City, Gyeonggi-do, Republic of Korea; 2 Ewha Medical Academy, Ewha Womans University Medical Center, Seoul, Republic of Korea

**Figure:**
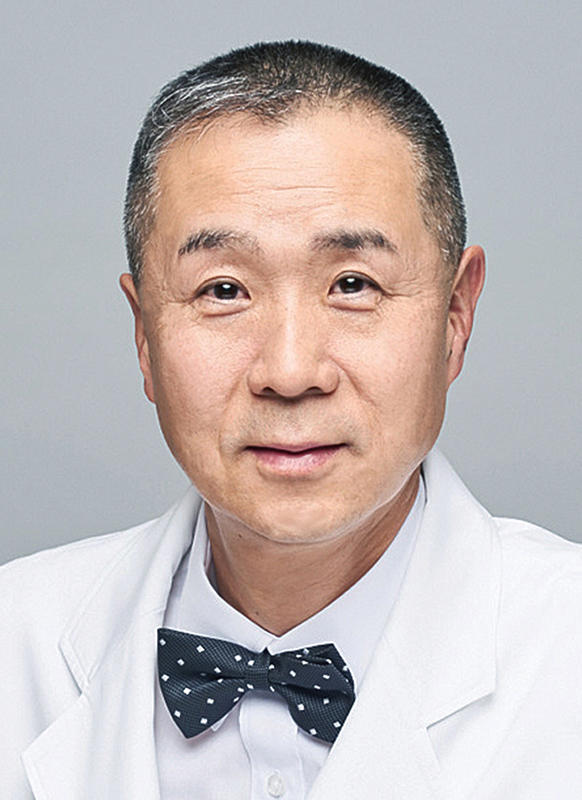
Kun Hwang, MD, PhD

According to the suggestions of theorists, ancient artists felt that the archaic smile represented the blessing of the gods for the actions of the figures portrayed. It is also thought that this smile reflected a state of ideal health and well-being. In Buddhist countries, most people think the Buddha's face looks merciful. In ancient Korea, Baekje sculptures exhibited distinct characteristics of warmth and softness and used relaxed poses to convey friendliness and an air of pleasantness that is rarely found in other traditions of Buddhist sculpture. 1 2

However, contrary to our expectations, there has been an “ugly Buddha,” as described in the “Sutta-Nipata.” 3

At that time there was born to the first wife of King Prasenajit a daughter who was named Vajra. The child's features and complexion were exceedingly ugly and her flesh was as rough as the hide of a horse. Her hair was as coarse as a wild horse's mane, and the king and queen regarded her with horror, kept her hidden away in the palace, and allowed no one to see her.

The king found an impoverished noble who is unmarried and this man became King's son in law (prince). This prince was ordered by the King to lock the gates and take the key with him to hide his ugly princess.

**Fig. 1:**
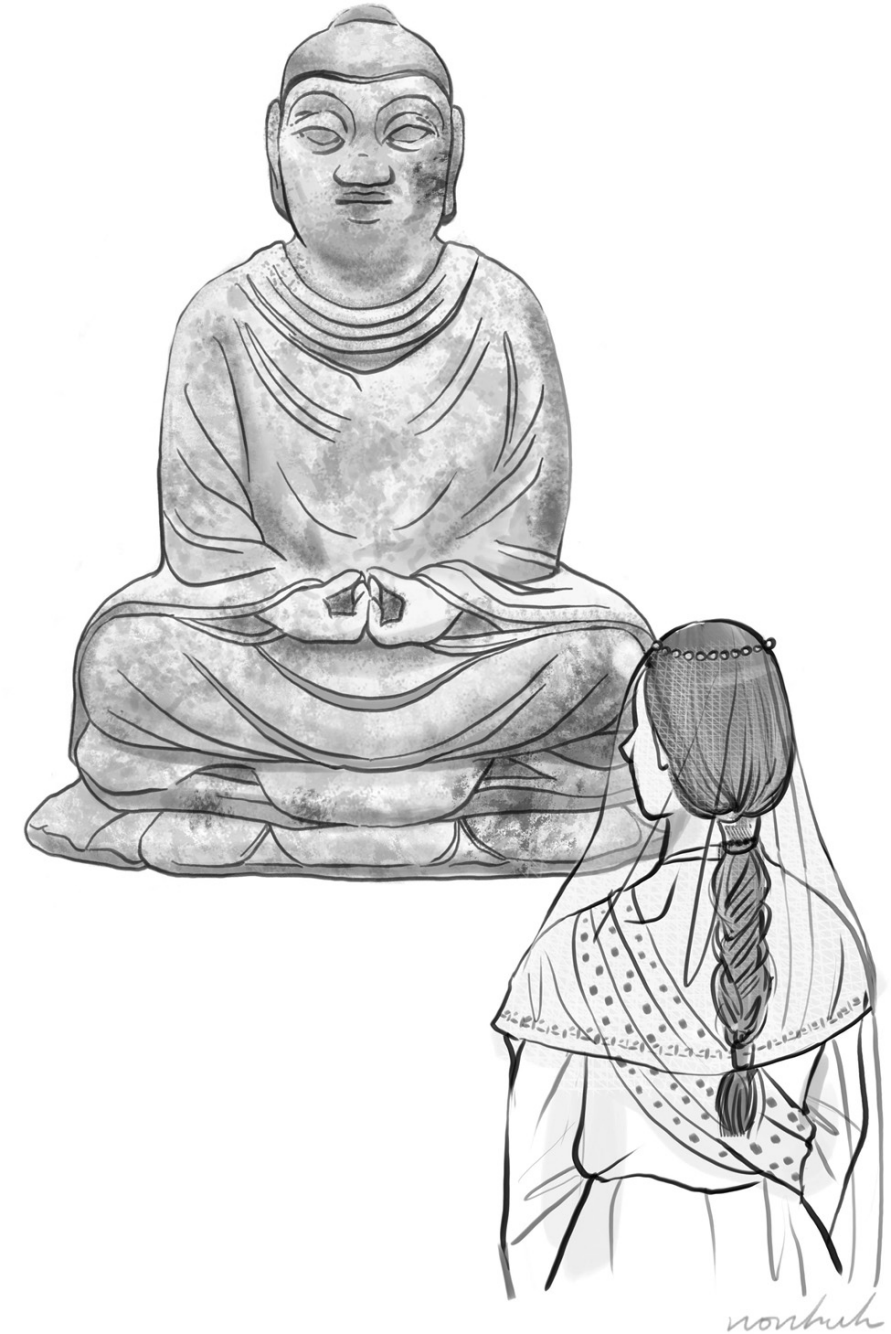
Pratyekabuddha fixed an ugly princess by making her a beauty. Illustration by Hye Won Hu, MA (1986–).

In the deep castle, the princess was thinking: “Because of what former sinful deeds have I been born so ugly and am obliged to live in this dark house, never seeing the sun or man, never meeting other people, and subjected to such suffering?” Bowing to the Buddha from afar, she prayed mentally: “Lord, compassionately show me my former existences, I beseech you.” The Buddha immediately knew her ardent desire and appeared to her, showing only his flame-like tuft of hair or usnisa. When the princess saw this, she rejoiced greatly, had faith, and her mind became totally pure. Because her mind had become pure, her hair became soft and black. Then the Enlightened One manifested his face to her. When the princess saw this, she rejoiced greatly and because of her faith her face became beautiful and lovely, and its coarseness and ugliness disappeared. Likewise, her ugly complexion of the body disappeared, and she became more beautiful than a daughter of the gods. When the Lord had explained the Dharma to her, her sins were purified and she attained the fruit of a streamwinner ( Fig. 1 ).

When her husband returned home, at first he could not recognize her because his ugly wife had just changed into a beauty. They told the king that through the compassionate blessing of the Buddha, the princess had become beautiful and lovely.

The King knelt onto his right knee and asked the Buddha: “Lord, by virtue of what former good deeds was this daughter of mine born in a high caste and with great wealth, and by reason of what sinful deeds was she born ugly, her hair and skin like those of an animal? What were the causes of this?”

The Buddha replied: “It is because of both virtuous and evil deeds done in the past that she was taken on both these forms. In times long past there was a householder in the land of Benares who was wealthy beyond comparison. All his property he placed at the disposal of a Pratyekabuddha. This Pratyekabuddha's body was rough and ugly and his complexion was very bad. Every day when he came to the householder's home, the householder's little daughter would insult him and ask her father what he was doing with such an ugly person. Upon a certain occasion, the Pratyekabuddha came to the house, accepted offerings, and prayed that he might obtain Nirvana. Then, he performed wonders for the householder and his friends. Descending from the sky, he entered the householder's house and the householder was delighted. The little girl, seeing all this, regretted having insulted the Pratyekabuddha and asked for his forgiveness: “Noble One, I confess my sin of insulting you with silly, had words. May I be forgiven?” The Pratyekabuddha told her: “You are forgiven.”

“This daughter of yours was that girl. At that time, because she insulted the noble Pratyekabuddha with a sinful mind, she has suffered from ugliness. When she saw his wonders, repented, and confessed and made offerings, she became beautiful and endowed with a good mind. It is because she made offerings to a Pratyekabuddha that in whatever place she is reborn she will be born in a high caste with wealth and in the end will be perfectly liberated.”] 3

A Pratyekabuddha is an individual who independently achieves liberation without the aid of teachers or guides and without teaching others to do the same. In Buddhism, health is viewed as the harmonious interplay of all these forces in accordance with the 8-fold path of Buddhism. Consequently, disease is considered as an expression of disharmony, which prevents an individual from living in a holistic manner. According to the Thai Buddhist approach, disease may also result from surrogate agents such as ghosts, demons, jinn, or ancestors, who may possess or afflict the person. In addition, the concept of karma contributes to an individual's state of health and illness. Health and disease are interpreted as the effects of positive or negative karma that has accumulated from previous lives. 4

Nowadays, in Thailand, a Buddhist country, interest in cosmetic surgery and the ethics of beauty is growing. 5 Many Thai women undergo cosmetic surgery to “look more Caucasian.” For many young and older middle-class Thai women who have assimilated Western aesthetic values, the aim is to create a body that is not viewed as being physically deficient. 6

Like the ugly Pratyekabuddha who fixed an ugly woman by making her a beauty, we plastic surgeons fix faces and bodies that are not viewed as being physically deficient.
